# Expression of a Cutinase of *Moniliophthora roreri* with Polyester and PET-Plastic Residues Degradation Activity

**DOI:** 10.1128/Spectrum.00976-21

**Published:** 2021-11-03

**Authors:** Laura Vázquez-Alcántara, Rosa María Oliart-Ros, Arturo García-Bórquez, Carolina Peña-Montes

**Affiliations:** a Tecnológico Nacional de México/IT Veracruz, Unidad de Investigación y Desarrollo en Alimentos, Veracruz, México; b Instituto Politécnico Nacional, Escuela Superior de Física y Matemáticas, UPALM, Mexico City, México; Broad Institute

**Keywords:** cutinase, *Moniliophthora roreri*, polyester degradation

## Abstract

Cutinases are enzymes produced by phytopathogenic fungi like Moniliophthora roreri. The three genome-located cutinase genes of M. roreri were amplified from cDNA of fungi growing in different induction culture media for cutinase production. The *mrcut1* gene was expressed in the presence of a cacao cuticle, while the *mrcut2* and *mrcut3* genes were expressed when an apple cuticle was used as the inducer. The sequences of all genes were obtained and analyzed by bioinformatics tools to determine the presence of signal peptides, introns, glycosylation, and regulatory sequences. Also, the theoretical molecular weight and pI were obtained and experimentally confirmed. Finally, cutinase 1 from *M. roreri* (MRCUT1) was selected for heterologous expression in Escherichia coli. Successful overexpression of MRCUT1 was observed with the highest enzyme activity of 34,036 U/mg under the assay conditions at 40°C and pH 8. Furthermore, the degradation of different synthetic polyesters was evaluated; after 21 days, 59% of polyethylene succinate (PES), 43% of polycaprolactone (PCL), and 31% of polyethylene terephthalate (PET) from plastic residues were degraded.

**IMPORTANCE** Plastic pollution is exponentially increasing; even the G20 has recognized an urgent need to implement actions to reduce it. In recent years, searching for enzymes that can degrade plastics, especially those based on polyesters such as PET, has been increasing as they can be a green alternative to the actual plastic degradation process. A promising option in recent years refers to biological tools such as enzymes involved in stages of partial and even total degradation of some plastics. In this context, the MRCUT1 enzyme can degrade polyesters contained in plastic residues in a short time. Besides, there is limited knowledge about the biochemical properties of cutinases from *M. roreri.* Commonly, fungal enzymes are expressed as inclusion bodies in E. coli with reduced activity. Interestingly, the successful expression of one cutinase of *M. roreri* in E. coli with enhanced activity is described.

## INTRODUCTION

Cutinases (EC 3.1.1.74) are carboxylic ester hydrolases that carry out the hydrolysis of a lipid polymer called cutin, a structural component of plants (1, 2). Cutin is a lipidic polymer of the plant cuticle formed by hydroxylated epoxy fatty acids of 16 and 18 carbons linked by ester bonds (3). Some bacteria such as Bacillus subtilis, *Citrobacter*, Enterobacter, and actinomycetes such as Mycobacterium and *Streptomyces*; yeasts such as Candida tropicalis; and many phytopathogenic fungi from the genera Aspergillus, Fusarium, *Trichoderma*, and *Moniliophthora* has been shown to produce cutinases (4–8). More than one cutinase gene has been found in phytopathogenic fungal genomes. For example, Fusarium solani possesses two, Monilia fructicola contains four, and Moniliophthora roreri has three putative cutinase genes in their genome (8–12). In Aspergillus nidulans, four cutinase genes are located in the genome, two of which have been studied and characterized: ANCUT1 with esterase activity and ANCUT2 with additional cutinase activity (9, 10). In fact, it has been demonstrated that recombinant cutinases from A. nidulans can efficiently degrade polyesters (13).

Moniliasis disease of cacao crops is caused by the biotrophic or hemibiotrophic fungus M. roreri. To destroy the plant tissue causing necrosis, the production of several enzymes and toxins is essential. In *M. roreri*, several enzymatic activities in media with different carbon sources have been previously reported (12).

Cutinases have various applications in the food industry, particularly in oils, fats, and flavors (1). Besides, they have been used in other industries such as detergents and fur (14). They have also been valuable for degrading contaminating compounds such as polyesters and pesticides (14, 15). Plastics are long-persisting synthetic polymers like polyesters. Plastic pollution is exponentially increasing; even the G20 has recognized an urgent need to implement actions to reduce marine litter, especially marine plastic litter and microplastics (16). The amount of plastic waste before the coronavirus disease 2019 (COVID-19) pandemic was 4.8 million to 12.7 million metric tons; nevertheless, due to the contingency and the new use of N95 masks made of polypropylene (PP) and polyethylene terephthalate (PET) and in the same way the use of surgical gloves, there is a probability that the number of microplastics will increase in aquifer mantles (17). Chemical and physical methods have been used for plastic material degradation; however, some polymers are resistant to these methods and are harmful to the environment (18). A promising emergent solution to plastic pollution is the enzymatic degradation of polyesters contained in recalcitrant plastics like PET or even in biodegradable plastics like polycaprolactone (PCL) and polyethylene succinate (PES) (19–23). Biological treatments involving microorganisms and enzymes for plastic waste degradation are being evaluated (18, 19, 24).

Cutinases are valuable enzymes, which are emerging tools for the decontamination of plastic residues (24). Recently, a synergistic system composed of Microbacterium oleivorans JWG-G2 and the Thermobifida fusca cutinase was used to degrade bis(hydroxyethyl)terephthalate (BHET) and a PET film, obtaining terephthalic acid (TPA) and terephthalate mono(2-hydroxyethyl) (MHET) as a result (16). This work describes the heterologous expression of a cutinase of the cacao phytopathogenic fungus *M. roreri* in Escherichia coli, besides their characterization and polyester degradation capacity.

## RESULTS

### Isolation of cutinase genes.

**FIG 1:**
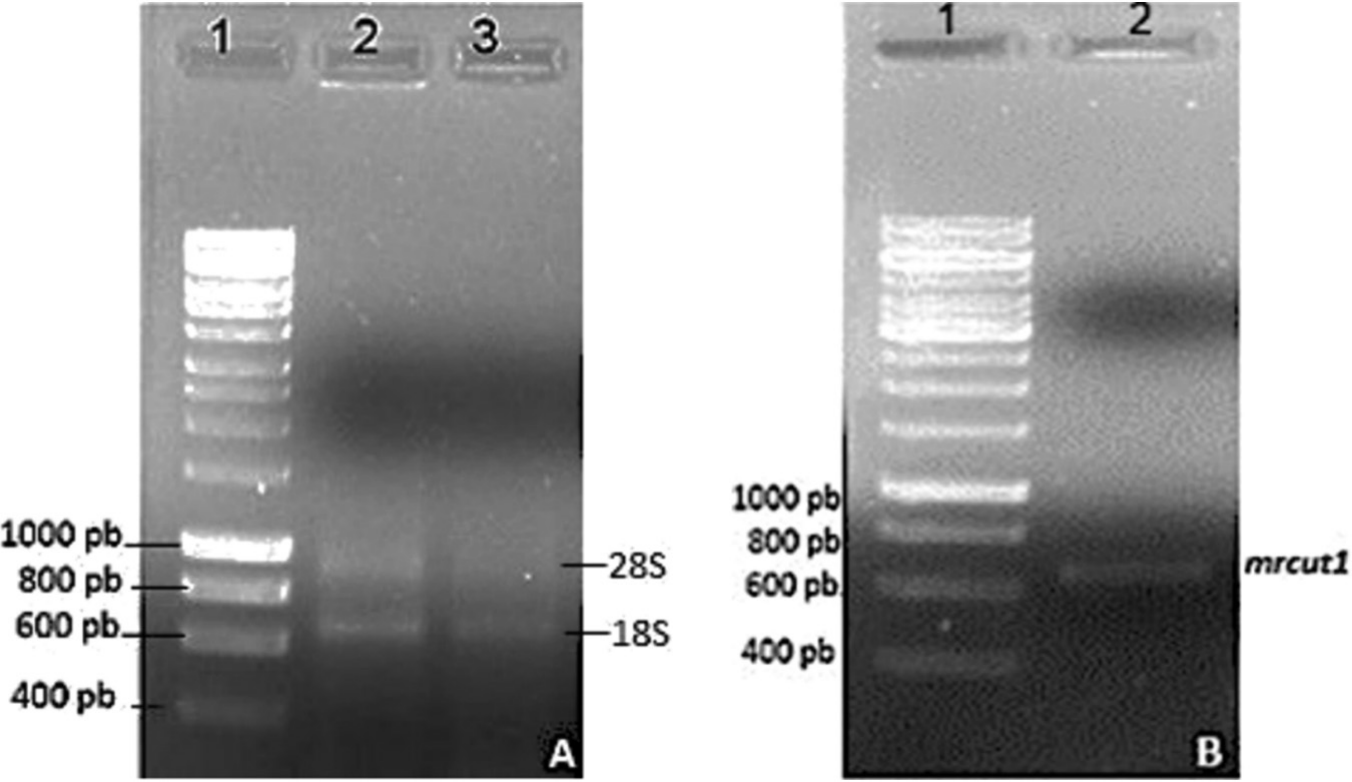
Isolated RNA and cutinase *mrcut1* gene amplified from mRNA in a 1% agarose gel. (A) 18S and 28S mRNAs from mycelia grown in the presence of different inductors. (B) Isolated cutinase gene.

The isolated ribonucleic acid (RNAs) of *M. roreri* induced with apple cuticle, cacao cuticle, or olive oil are shown in Fig. 1A. The *M. roreri* genome encodes three putative cutinases. Subsequently, complementary deoxyribonucleic acid (cDNA) was synthesized from the different extracted RNAs and used as a template for reverse transcription-PCR (RT-PCR) with cutinase-specific primers, and the *mrcut1* cutinase gene was expressed in minimum medium that contained a cacao inducer. The amplicon size obtained was approximately 600 bp, which was expected by the theoretically calculated value (558 bp) without introns (Fig. 1B). The genes encoding the MRCUT2 and MRCUT3 enzymes expressed in minimum medium with apple cuticle have lengths of 500 and 600 bp, respectively; these molecular sizes are not consistent with the theoretically expected deoxyribonucleic acid (DNA) size, probably due to the presence of introns. Next, the *mrcut1* gene for the cutinase ESK97883.1 (GenBank) was chosen for subsequent experiments with UniProt accesion number WG66_16246.

**FIG 2:**
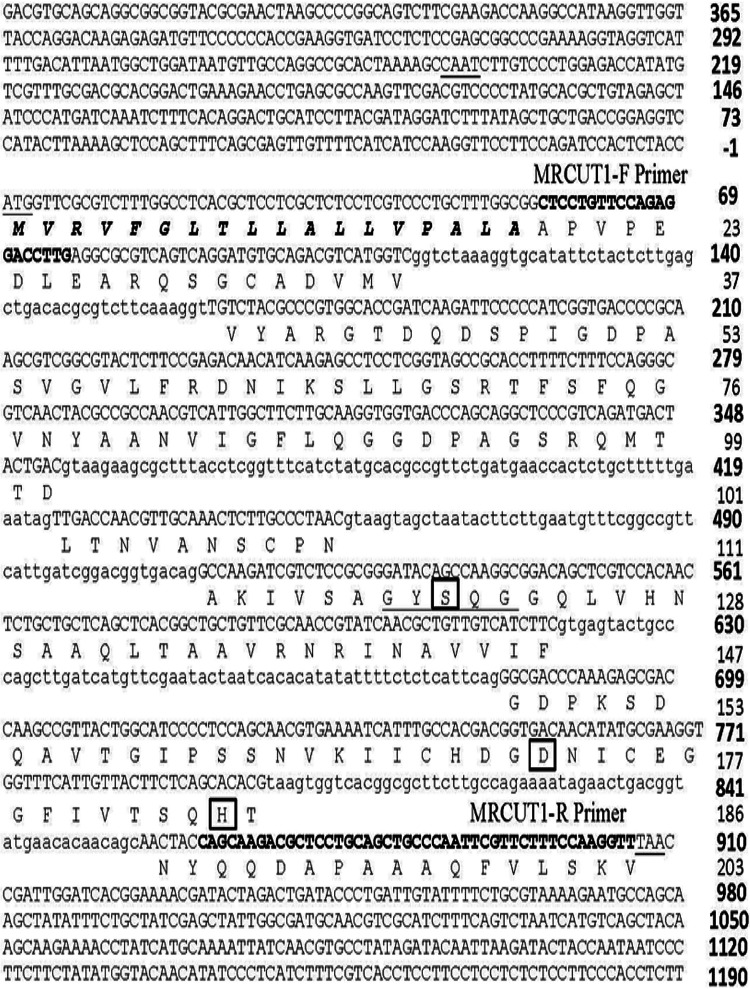
Alignment of the amino acid and DNA sequences of the MRCUT1 cutinase. Sequences are numbered on the right; boldface numbers correspond to the deoxyribonucleic acid (DNA) sequence, and the protein sequence is not in boldface type. The designed forward and reverse primers are marked in boldface uppercase type. Introns are in lowercase letters. Start and stop codons are underlined. The catalytic triad’s amino acids (Ser120, Asp172, and His185) are enclosed, highlighting the conserved pentapeptide sequence. The amino acid signal peptide sequence is shown in boldface cursive letters.

Data from the bioinformatic analysis of the *mrcut1* gene are displayed in Fig. 2. The cutinase gene has a signal peptide from amino acids 1 to 18, containing five introns. The mature protein has 185 amino acids with a theoretical molecular weight of 19.28 kDa and a pI of 5.17. The canonical catalytic triad from all carboxylic acid ester hydrolases is observed; it is formed by serine 120, which is contained in the consensus pentapeptide of serine hydrolases (G-Y-S-Q-G); aspartic acid 172; and histidine 185. It is important to mention that the Kozak consensus sequence for a translation initiation site was observed for the *mrcut1* gene, which means that a purine (A) is present 3 bases upstream of the start codon (AUG) at positions 1 to 3, followed by a guanine (G) (25) (Fig. 2). Moreover, the glycosylation protein prediction analysis displayed no N-glycosylated sites and only one O-glycosylated site at serine 95.

**FIG 3:**
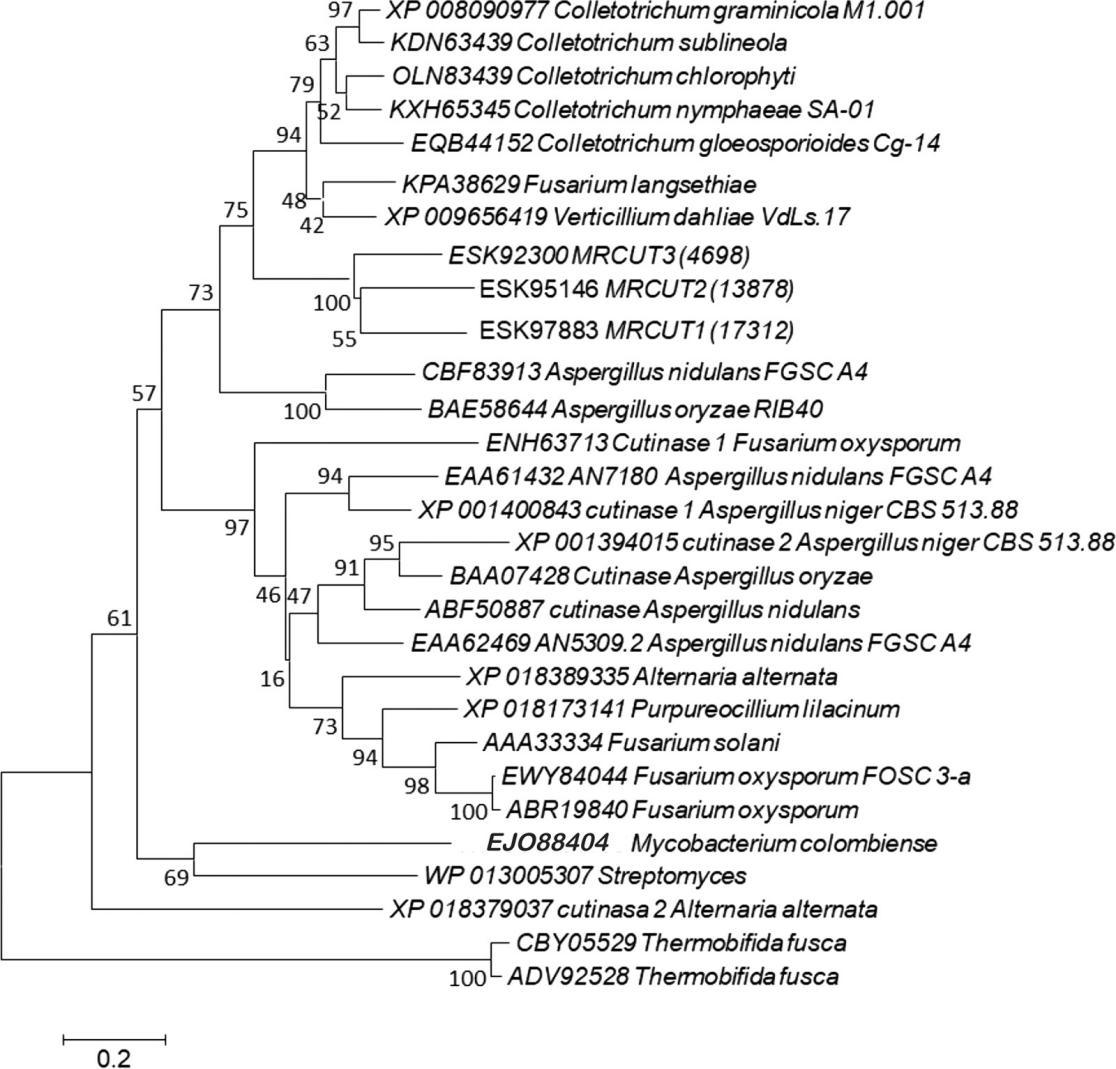
Phylogenetic tree of cutinase genes from *M. roreri* and other fungi. Evolutionary history is inferred from the neighbor-joining method (56). The optimal tree with the sum of the length of the branch is 9.31922869. The percentage of repetitions in which the associated taxa are grouped in the bootstrap test (10,000 repetitions) of trees is shown next to the branches (48).

The evolutionary relationship between *M. roreri* cutinases and others from different microorganisms was investigated by constructing a phylogenetic tree using the cutinase sequences retrieved from GenBank. The results revealed that all *M. roreri* cutinases are clustered in the same branch (Fig. 3).

### Overexpression of the *mrcut1* gene in E. coli.

The *mrcut1* gene was successfully inserted into the pET22b vector. Next, pET22*mrcut1* was purified and used for the transformation of E. coli BL21. PCR with T7 universal primers corroborated the presence of the insert. The amplicons obtained showed a size of between 750 and 1,000 bp, corresponding to the theoretical one (890 bp). After bioinformatics analysis, the amplicon sequence displayed high similarity (98%) with the cutinase gene of *M. roreri* MCA 2997 in the NCBI database under accession number ESK97883. The *mrcut1* gene was also in the correct open reading frame.

**FIG 4:**
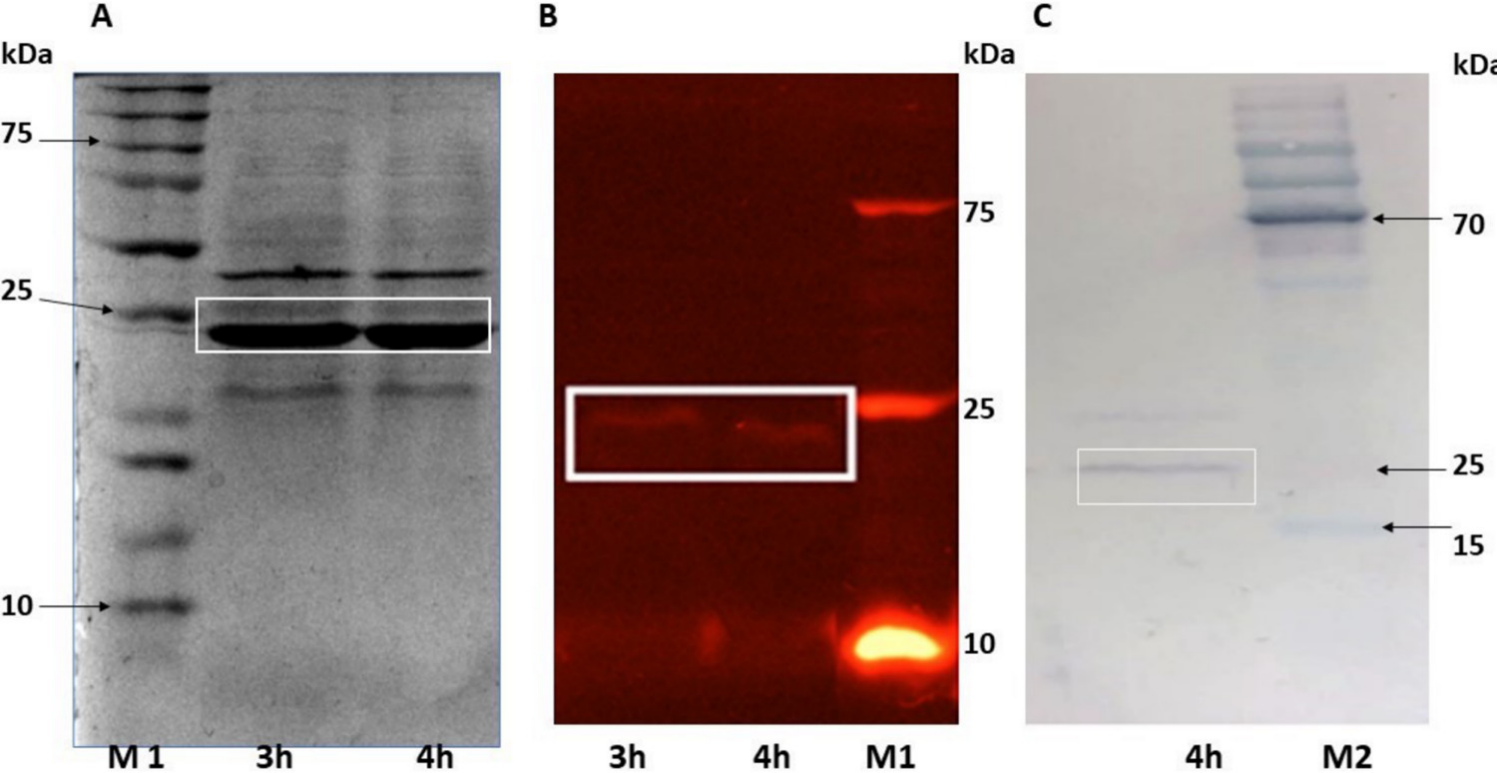
Intracellular protein profile in SDS-PAGE gels of recombinant E. coli containing the *mrcut1* gene. (A) Polyacrylamide gel with Coomassie staining; (B) zymogram; (C) Western blotting. M1, molecular weight marker (Kaleidoscope; Bio-Rad); 3 h and 4 h, intracellular extracts of recombinant E. coli after 3 and 4 h of induction; M2, molecular weight marker (PageRuler prestained; Thermo Scientific). Shown is an intense band of approximately 23 kDa, which is boxed.

A 4-h induction with IPTG (isopropyl β-d-1-thiogalactopyranoside) was done, and the protein profile after cell breakage was determined by SDS-PAGE (Fig. 4A). One intense band of approximately 23 kDa was observed, with *in situ* activity detected by zymography (Fig. 4B) and confirmed by Western blotting (Fig. 4C).

**FIG 5:**
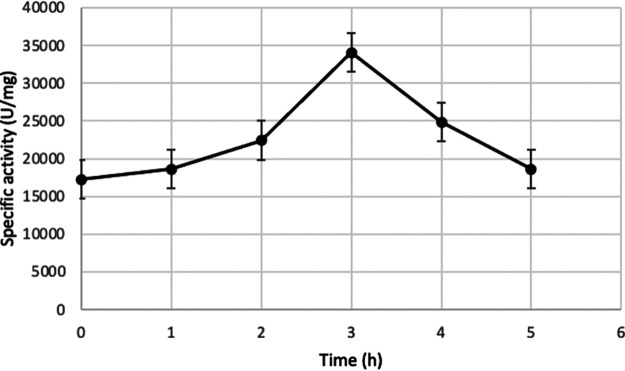
Induction kinetics of MRCUT1 expression. Specific activity was evaluated using PNA as a substrate. Obtained values are the means from three replicates.

**FIG 6:**
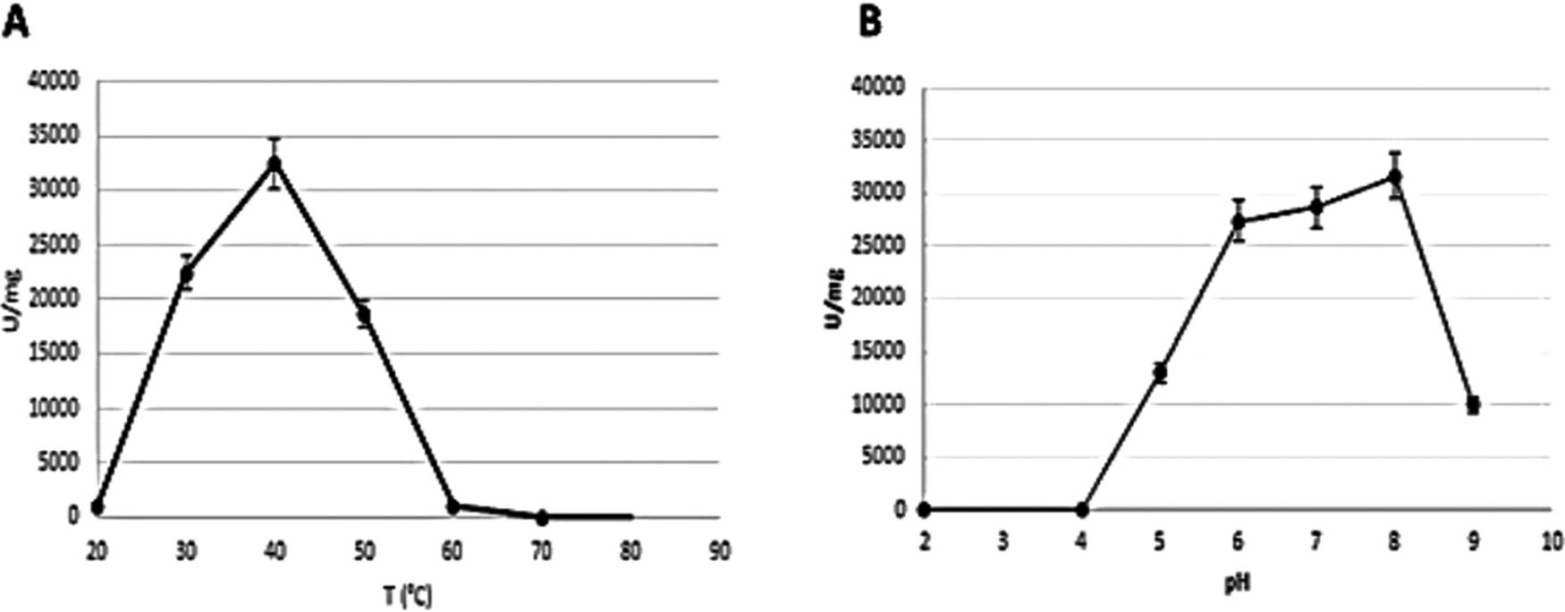
Dependence of enzymatic activity of MRCUT1 on temperature (A) and pH (B). Results are the means from three replicates.

MRCUT1 production was monitored for 5 h, and its activity was quantified by the hydrolysis of *p*-nitrophenyl acetate (p-PNA) (Fig. 5). A maximum peak at 3 h of induction was observed, which was established as the optimal time of induction of the recombinant protein MRCUT1. The highest specific activity under the evaluated conditions was 34,036 U/mg. The optimum temperature of MRCUT1 was 40°C, and the optimum pH was 8 under the evaluated conditions (Fig. 6).

### Polyester degradation.

**FIG 7:**
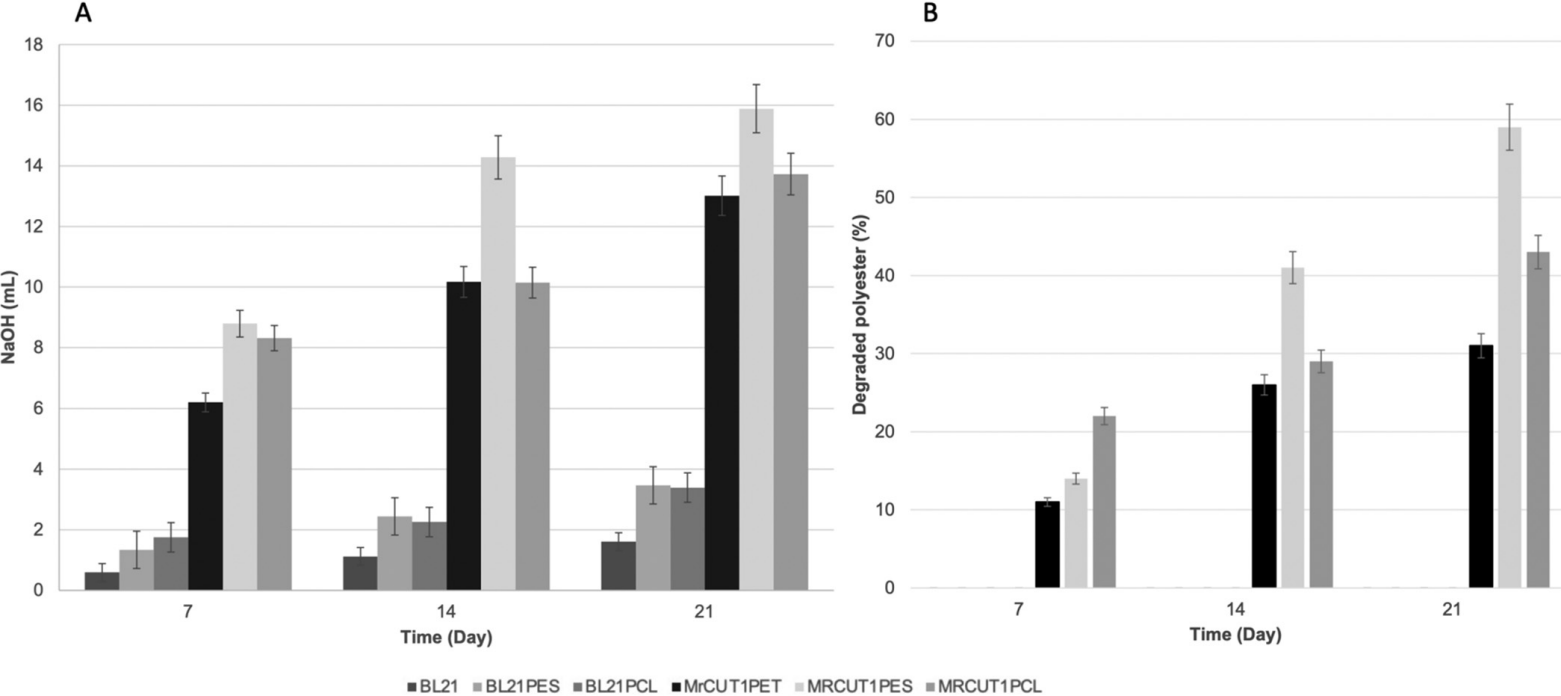
Kinetics of degradation of polyesters by MRCUT1. (A) Degradation measured by titration of fatty acids. (B) Percent degradation of polyesters determined by weight loss. Results are the means from three replicates. BL21, samples of an E. coli BL21 intracellular extract; BL21PES, samples of an E. coli BL21 intracellular extract with PES; BL21PCL, samples of an E. coli BL21 intracellular extract with PCL; MRCUT1PET, samples of an extracellular extract of recombinant E. coli BL21 containing the *mrcut1* gene with PET; MRCUT1PES, samples of an extracellular extract of recombinant E. coli BL21 containing the *mrcut1* gene with PES; MRCUT1PCL, samples of an intracellular extract of recombinant E. coli BL21 containing the *mrcut1* gene with PCL.

Polyester degradation was evaluated by two techniques, with similar results. The highest level of degradation was observed after 21 days when PES was used as the substrate: 43% as determined by weight loss and 16.2 ml by titration. In the PET bottles, 31% degradation was obtained by weight loss, and 13 ml was obtained by titration. In the PCL degradation assay, 59% was obtained by weight loss, and 13.91 ml was obtained by titration (Fig. 7).

**FIG 8:**
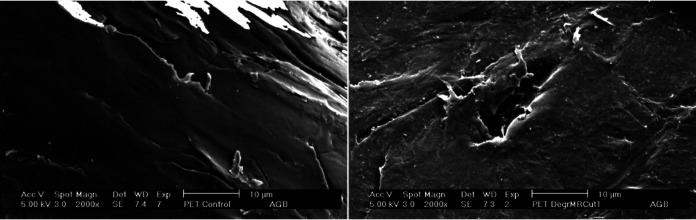
PET fragment micrograph. (A) Negative control for PET (without enzyme); (B) PET treated during 21 days with the enzyme MRCUT1.

Finally, a scanning electron microscopy (SEM) evaluation of PET films was done (Fig. 8). We detected the degradation of PET films treated with MRCUT1, which was observed as holes with scraped edges and eroded surfaces (Fig. 8B). In contrast, the surface looked smooth with the control PET, with only cracks belonging to the original PET (Fig. 8A).

## DISCUSSION

*M. roreri* contains several cutinase genes, as reported for other fungi like Aspergillus nidulans and Fusarium solani (24, 26). We found that the genome of *M. roreri* encodes three cutinases that are induced under different conditions. *mrcut1* is induced with cocoa cuticle. In contrast, *mrcut2* and *mrcut3* are induced with apple cuticle and olive oil, respectively. The results obtained in the presence of cocoa cuticles follow previous work that reported the transcriptional expression of the *mrcut1* gene in medium supplemented with cocoa pod shells (12). In the case of *mrcut2* and *mrcut3*, gene expression was not previously reported.

Interestingly, *M. roreri* cutinase expression is similar to that observed for other phytopathogenic fungi such as A. nidulans, whose cutinases are also differentially expressed; for example, *ancut2* was detected only in the presence of olive oil (9, 10). After gene isolation, the sequencing results displayed that the *mrcut1* gene has no introns. However, the *mrcut2* gene has retained 2 introns from 3, and *mrcut3* kept 1 from 5; this finding could be a consequence of alternative splicing, as has been observed for other enzyme genes (27). It would be interesting to clone the obtained *mrcut2* and *mrcut3* genes to establish the differences in their biochemical properties and reaction specificities.

The phylogenetic analysis displayed that all cutinases of *M. roreri* were grouped in the same clade, with MRCUT1 and MRCUT2 being more related (55% similarity). Moreover, both enzymes contain almost the same numbers of amino acids (203 and 198, respectively), while MRCUT3 contains only 127 amino acids. It is important to mention that all *M. roreri* cutinases are related to other fungal cutinases that contain 4 cysteines, such as the cutinase from A. nidulans under GenBank accession number CBF83913.1.

The enzyme MRCUT1 with GenBank accesion number KTB31184.1 was chosen for cloning and was successfully overexpressed in E. coli, as shown in Fig. 4A as an intense wide band of approximately 23 kDa; this was confirmed by the *in situ* esterase activity with MUF (4-methylumbelliferone)-butyrate as the substrate (Fig. 4B). Additionally, recombinant cutinase expression was confirmed by Western blotting with anti-His tag antibodies (Fig. 4C). These results agree with the theoretical molecular weight of the protein of interest (21.8 kDa) after the correct processing of the *pelB* signal peptide.

Once overexpression was corroborated, kinetic MRCUT1 production was determined. The maximum specific activity under the evaluated conditions was 34,036 U/mg after 3 h of induction. Interestingly, this value is higher than those of other fungal cutinases overexpressed in E. coli, such as the cutinase of Thermobifida fusca (167.4 U/mg) considering *p*-NPA as the substrate, even after improving T. fusca cutinase expression was done (21, 28, 29). Su and collaborators obtained 1,063.5 U/ml after cloning the truncated version of the extracellular T. fusca cutinase without the signal peptide, obtaining higher specific activity and the extracellular release of the enzyme (30). In this work, we have cloned MRCUT1 without a signal peptide, also with high specific activity, but most of the enzyme, as expected, was intracellularly located. The correct processing of the *pelB* signal peptide and folding of the enzyme can contribute to the observed high enzyme activity. The finding is also successfully unexpected for a eucaryotic cutinase gene expressed in a procaryotic system. Fungal cutinases can be glycosylated, which impedes enzyme aggregation and improves its stabilization; however, for MRCUT1, only one predicted glycosylation site was found (serine 95). Besides, the activity in the recombinant enzyme was not committed, which indicates that this modification is not necessary for MRCUT1, and it is probably not present in the native enzyme (21).

High enzymatic activity is observed even under no-induction conditions; this follows other reports where the expression of recombinant enzymes under no-induction conditions has been observed, known as leakage phenomena (21, 29, 30–31). Another possibility is that cutinases can partially hydrolyze the phospholipids in the E. coli membrane (31).

MRCUT1 has an optimum temperature of 40°C and an optimum pH of 8, similar to the results obtained for cutinases of other filamentous fungi such as Fusarium solani, Alternaria brassicicola, and Aspergillus nidulans (9, 11, 32). Fungal cutinases having six cysteines are thermostable; in this case, as shown in Fig. 2, MRCUT1 contained four cysteines forming two disulfide bonds, and it is not thermostable (9) (Fig. 6).

Finally, in recent years, enzymatic modification of synthetic polymers has been increasingly studied because of the prolonged environmental persistence of these materials (33). Replacing harsh chemicals with enzymes is an environmentally friendly technique for polymer waste treatment (34). Biodegradability is a crucial characteristic of aliphatic polyester materials (PES and PCL), which are widely used in injection-molded products and the pharmaceutical and biomedical industries and recently for 3D (three-dimensional) printing materials; however, research about the biocatalytic degradation of these materials is scarce (23, 35). On the other side, aromatic polyesters (PET) are recalcitrant, and their residue accumulation is increasing at an alarming rate (36). We explored the possibility of using recombinant MRCUT1 for the degradation of PES, PCL, and PET. Our results demonstrate that recombinant MRCUT1 can efficiently hydrolyze biodegradable polyesters like PES and PCL; however, it can also degrade long-persistence polyesters like PET in residues of water storage bottles in 21 days. After 6 days, PES films can be almost totally degraded (95%) by recombinant F. solani cutinase but cannot be degraded by polyhydroxybutyrate depolymerase from Aspergillus fumigatus (23, 37). MRCUT1 cutinase can degrade 43% after 21 days. For PCL, biodegradation is slower because of its high hydrophobicity and degree of crystallinity; MRCUT1 degrades 59% after 21 days, and similar results were obtained in compost where the fungus A. fumigatus was the most abundant microorganism found (22). A recent study displayed PCL film degradation after using Candida antarctica lipase and F. solani cutinase; it has been found that weight loss reached 87.56% and 80.8%, respectively, after 72 h (38).

Furthermore, 31% degradation of PET film, which is highly crystallized from commercial bottles, was obtained by weight loss after 21 days. It was corroborated by SEM images, which showed eroded surfaces and holes in samples treated with the recombinant cutinase MRCUT1. Compared with other enzymes that have demonstrated efficient PET film degradation, such as the *Ideonella sakaiensis* PETase (IsPETase), or cutinase from leaf-branch compost (LC-cutinase), the MRCUT1 cutinase has a promising capacity (39, 40). Those enzymes initially showed results similar to those of MRCUT1 and better results after improving protein engineering or reaction parameters (40–42). For example, at first, Ideonella sakaiensis had a PET film weight loss of less than 20% in 20 days, and recently, after using improved consensus protein engineering, the degradation activity for amorphous PET was increased by almost 40-fold in comparison with the wild type at 40°C in 24 h (40, 41). The LC-cutinase initially could degrade 1.45 mg in 24 h from a 20- to 25-mg PET film, and after using computer-aided enzyme engineering of LC-cutinase, an improvement of enzyme-catalyzed PET depolymerization to 90% conversion in less than 10 h was obtained (39, 42). We must take advantage of the fact that this recombinant enzyme is expressed in large quantities and with high activity in this expression system.

### Conclusions.

The mature cutinase MRCUT1 of *M. roreri* was successfully overexpressed in E. coli with enhanced specific activity even under no-induction conditions. MRCUT1 is a new alternative enzyme for the efficient degradation of films of plastic residues from aliphatic polyesters (PES and PCL) and aromatic polyesters (PET) contained in plastic bottles, indicating its environmental applicability for plastic reduction in an eco-friendly manner. New enzymes are needed for plastic depolymerization; this work further acknowledges polyester degradation by cutinases. It is important to mention that this is the first report of polyester film degradation by MRCUT1 cutinase. Consequently, the enzyme MRCUT1 can be a suitable candidate for future protein engineering, looking for high-level and short-time eﬃcient polyester degradation under extreme conditions. Besides, reaction parameters such as pH, temperature, enzyme quantity, substrate availability, or the addition of surfactants must be evaluated for better polyester degradation.

## MATERIALS AND METHODS

### Microorganisms.

*M. roreri* was previously isolated from cacao pods (12). E. coli BL21 [F^−^ *ompT hsdS*_B_(r_B_^−^ m_B_^−^) *gal dcm* (DE3)] and plasmid pET22b^+^ from the pET system (Novagen) were purchased.

### Microorganism growth.

Liquid minimum induction medium (50 ml) in 250-ml Erlenmeyer flasks was inoculated with 1 × 10^8^ spores/ml of *M. roreri*. The medium contained salts (KNO_3_ at 6 g/liter, K_2_HPO_4_ at 1.5 g/liter, MgSO_4_·7H_2_O at 0.5 g/liter, FeSO_4_·7H_2_O at 0.002 g/liter, ZnSO_4_·7H_2_O at 0.002 g/liter, and MgCl_2_·7H_2_O at 0.002 g/liter), dextrose (0.5%), and an induction source at 1% (apple cuticle, cacao cuticle, or olive oil). Cultures were incubated at 27°C in an orbital shaker at 300 rpm for 4 days.

E. coli BL21 was inoculated in Luria-Bertani (LB) medium. Briefly, 20 μl of E. coli cultures was added to obtain an optical density at 600 nm (OD_600_) of 0.9 to 10-ml assay tubes containing 5 ml of LB broth. Cultures were incubated at 37°C in an orbital shaker at 250 rpm for 12 h.

### Cuticle extraction.

The cuticle was obtained according to the method described previously by Castro-Ochoa et al. (9).

### RNA isolation and cDNA synthesis.

Fresh mycelium from *M. roreri* was frozen by immersion in liquid nitrogen, ground to a powder in a prechilled mortar, and stored at −80°C until RNA extraction. RNA was extracted with TRIzol according to the provider’s protocol (Invitrogen), treated with DNase (Thermo Fisher) according to the manufacturer’s instructions, and used as the template for cDNA synthesis with the cDNA cycle kit according to manual instructions (Thermo Fisher). The reaction mixture was incubated for 5 min at 25°C for cDNA synthesis and then for 60 min at 42°C and 5 min at 70°C.

### Primers and PCR conditions for cutinase gene amplification.

The three cutinase genes of *M. roreri* were amplified with *Taq* PCR polymerase (Invitrogen) and three pairs of the designed primers described in Table 1. EcoRI/NotI restriction sites were introduced into each cutinase gene of *M. roreri.* The following program was used: 1 cycle at 94°C for 5 min followed by 35 cycles of 94°C for 40 s, 55°C for 40 s, and 72°C for 1 min; 1 cycle at 72°C for 10 min; and, finally, a storage step at 4°C.

**TABLE 1:** Primer sequences for cutinase gene amplification by PCR

Primer	Sequence (5′–3′)
mrcut1F	GCCGAATTCAGCTCCTGTTCCAGAGGACCTTG
mrcut1R	CAATTCGTTCTTTCCAAGGTTGCGGCCGCTCA
mrcut2F	GCCGAATTCGAGCTCCCGTTGAGCGCAGAGCCACCG
mrcut2R	TAACGCCGCTGTGATTTTTGTAGGTGCGGCCGC
mrcut3F	GCCGAATTCAGGCCCCCTTGGCTGAACGAGCAGAAT
mrcut3R	GGCAAAATTCGTTGTGTCAAAGTTGGCGGCCGCTCA

### Bioinformatic tools.

According to the provided instructions, amplicons were gel purified with a DNA clean and concentrator kit (Zymo Research). Purified PCR products were sequenced at the USSDNA at Biotechnology Institute (UNAM) in a PE Applied Biosystems 121 automated DNA sequencer (model 3130xl).

The internal transcribed spacer (ITS) region and the three isolated genes were analyzed using the NCBI database (Basic Local Alignment Search Tool). Signal peptide analysis was performed using SignalP 5.0 software (43), and the presence of introns in the sequences was determined with the Multaline program (44). Glycosylation prediction analysis was done with the NetNGlyc 1.0 server and the NetOGlyc 4.0 server (45, 46). Restriction analysis was performed with Nebcutter software (47). Phylogenetic analysis was performed with MEGA 6 with 1,000 replicates (48). The sequences used and their GenBank accession numbers (in parentheses) are cutinase from Colletotrichum graminicola (XP_008090977), cutinase from *Colletotrichum sublineola* (KDN63439), a putative cutinase from *Colletotrichum chlorophyti* (OLN83439), cutinase from *Colletotrichum nymphaeae* (KXH65345.1), cutinase from *Colletotrichum gloeosporioides* (EQB44152.1), cutinase from Verticillium dahliae (PA38629), cutinase from Fusarium *langsethiae* (KPA38629.1), cutinase from *M. roreri* MRCUT3 (ESK92300.1), cutinase from *M. roreri* MRCUT2 (ESK95146), cutinase from *M. roreri* MRCUT1 (ESK97883.1), a putative cutinase from Aspergillus nidulans (CBF83913), protein from Aspergillus oryzae (BAE58644), cutinase 1 from Fusarium oxysporum (ENH63713), a hypothetical protein from Aspergillus nidulans (EAA61432), cutinase 1 from Aspergillus niger (XP_001400843.1), cutinase 2 from Aspergillus niger (XP_001394015), cutinase from Aspergillus oryzae (BAA07428), cutinase from Aspergillus nidulans (ABF50887.1), a hypothetical protein from Aspergillus nidulans (EAA62469), cutinase 1 *Alternaria alternata* (XP_018389335), cutinase from Purpureocillium lilacinum (XP_018173141), cutinase from Fusarium solani (AAA33334), cutinase 3 from Fusarium oxysporum (EWY84044), cutinase from Fusarium oxysporum (ABR19840), cutinase from Mycobacterium colombiense (EJO88404), cutinase from Streptomyces scabiei (WP_013005307.1), cutinase from *Alternaria alternata* (XP_018379037), and cutinase from Thermobifida fusca (CBY05529). These sequences were aligned using ClustalW (49).

### Expression in Escherichia coli.

**(i) Cloning of the *mrcut1* gene in the pET22b vector.** The plasmid pET22b^+^ and the *mrcut1* cutinase gene were digested with NotI and EcoRI restriction enzymes (Thermo Fisher) and then ligated with T4 ligase (Thermo Fisher) according to the manufacturer’s instructions to obtain the pET-*mrcut1* recombinant vector.

**(ii) E. coli BL21 transformation.** Competent E. coli BL21 cells were elaborated as described by provider in the Novagen pET expression system manual, transformed by a chemical method with CaCl_2_, and cultured on LB-ampicillin agar plates (50). Ampicillin-resistant colonies were grown in liquid LB-ampicillin medium. The pET expression system manual describes colony PCR, which we have done with the specific primers described in Table 1 to corroborate the gene insertion.

**(iii) Induction of *mrcut1* gene expression.** The selected clones were grown in 5 ml of LB broth until an OD_600_ of 0.6 to 0.9 was reached (precultured cells). For induction, a 250-ml Erlenmeyer flask containing 50 ml of LB broth was inoculated with 200 μl of precultured cells and incubated at 37°C at 250 rpm until an OD_600_ of 0.9 was reached. Next, 0.4 mM IPTG (isopropyl β-d-1-thiogalactopyranoside) was added, and the culture was incubated under the same conditions as the ones described above until MRCUT1 expression was observed. An aliquot of 10 ml was taken each hour during the induction time and assayed for esterase activity.

**(iv) Enzymatic assays.** A qualitative assay was performed with a mixture of 75 μl of an α-naphthyl acetate (α-NA) solution (0.005 M α-NA, 25% acetone, and 50 mM sodium phosphate buffer [pH 7]) and 75 μl of the crude induced extract. The mixture was homogenized and incubated for 15 min at 37°C. After the reaction time, 75 μl of a Fast Red (FR) solution (0.008 M FR dissolved in 0.05% Triton X-100 and 50 mM sodium phosphate buffer [pH 7]) was added. Esterase activity was observed as a change in color to red-brown. Phosphate buffer (without enzyme) was used as the negative control (51).

Esterase activity quantification was done by using *p*-nitrophenyl acetate (p-PNA) as a substrate. A mixture of 25 μl of 1 mM *p*-NPA in ethanol, 200 μl of 50 mM sodium phosphate buffer (pH 7.5), and 25 μl of the pure enzyme or crude extract was homogenized and incubated at 37°C for 15 min, and the absorbance at 410 nm was measured every 3 min. Phosphate buffer without enzyme was used as a negative control. A standard curve of 25 to 200 μM *p*-nitrophenol was used to determine the enzymatic activity. An enzymatic activity unit (U) is defined as the amount that transforms 1 μmol of PNA per min.

**(v) Protein determination.** Proteins were quantified by the Bradford method according to the instructions provided by the manufacturer (Bio-Rad). A standard curve of 0.01 mg to 0.1 mg of bovine serum albumin (BSA) was used (52).

**(vi) SDS-PAGE and zymograms.** The SDS-PAGE technique was used to obtain the protein profiles of crude extracts in 14% acrylamide gels (53). Samples in sample buffer were boiled at 95°C for 5 min, loaded into each well, and run at 80 and 120 mV in a vertical chamber, and the gels were stained with Coomassie overnight. According to the protocol described previously by Brunelle and Green, the gels were stained and destained until bands were observed (54).

A zymogram was performed using 100 μM MUF (4-methylumbelliferone)-butyrate as a substrate to detect *in situ* enzymatic activity. After gel running, proteins were renatured by gel immersion in Triton X-100 (5%) for 30 min at 100 rpm and subsequent immersion in 50 mM sodium phosphate (pH 7) for 5 min. MUF-butyrate was added, and the mixture was left at room temperature for 10 min. The bands were observed under UV light (55).

**(vii) Western blotting.** After SDS-PAGE, proteins were transferred to a polyvinylidene difluoride (PVDF) membrane in a semidry transfer cell at 20 V for 1 h according to the provider’s protocol (Bio-Rad). A band corresponding to the recombinant enzyme was revealed by anti-His tag antibodies coupled to alkaline phosphatase (Thermo Scientific), which detect the His tag added at the enzyme C terminus.

**(viii) MRCUT1 cutinase purification.** Affinity purification was performed with a cobalt column (Clontech), according to the manufacturer’s protocol.

### Biochemical characterization.

Temperature and pH optima for the assayed conditions were determined by a spectrophotometric quantification method as described above. The effect of temperature on enzyme activity was assayed at 20°C, 30°C, 40°C, 50°C, 60°C, 70°C, and 80°C at pH 7.5 (0.1 M phosphate buffer) for 15 min with appropriate controls. The pH optimum was assayed during 30 min at 37°C in a pH range from 2 to 9 in the following buffers: 50 mM citrate (pH 2 and 4), 50 mM acetate (pH 5), 50 mM sodium phosphate buffer (pH 6 to 7), and 50 mM Tris-HCl (pH 8 to 9). Triplicates were performed for each reaction, and the values were corrected for the noncatalyzed hydrolysis of the substrate.

### Polyester degradation assay.

The polyester degradation ability of recombinant MRCUT1 cutinase was analyzed by three techniques, dry weight measurement, titration, and scanning electron microscopy (SEM).

**(i) Dry weight measurement.** First, circular films of approximately 3 mm in diameter and 1 mm thick were prepared, weighing around 10 mg. Polycaprolactone (PCL) and polyethylene succinate (PES) were melted at 80°C and compressed in a mold to obtain small films (22). For polyethylene terephthalate (PET), samples were used directly from commercial water bottles, and fragments were taken and cut into small pieces (5 mm^2^) weighing around 25 mg.

For the degradation assay, 0.05 g of small cut polyester pieces of PES, PCL, or PET was added to 1 ml of an enzyme solution (100 U/ml in 50 mM sodium phosphate buffer [pH 7.5]) in a 5-ml reaction vial. The vial was incubated at 37°C at pH 7.5 for 21 days, and a sample was taken each day for 7 days. The negative control was evaluated under the same conditions; in this case, only phosphate buffer was added to polyester. After the reaction time, the polyester was washed with ethanol and allowed to dry in an oven at 37°C overnight before dry weight measurement. PET was obtained from commercial water bottles, and PES and PCL were acquired from Sigma-Aldrich. After dry weight measurement, these samples were then used for SEM analysis as described below.

**(ii) Titration assay.** Upon hydrolysis, an aliquot of the reaction mixture was withdrawn and titrated with 0.01 N NaOH, and phenolphthalein was used as an indicator. The expenditure of milliliters of NaOH was recorded (39).

**(iii) Scanning electron microscopy.** An FEI Sirion XL30 SEM instrument with a field emission gun (FEG-SEM) was used to observe the polymeric films before and after the hydrolysis process with MRCUT1. A thin layer of Au was deposited on the polymeric samples to avoid charge accumulation during the SEM observations.
